# Downregulated developmental processes in the postnatal right ventricle under the influence of a volume overload

**DOI:** 10.1038/s41420-021-00593-y

**Published:** 2021-08-07

**Authors:** Chunxia Zhou, Sijuan Sun, Mengyu Hu, Yingying Xiao, Xiafeng Yu, Lincai Ye, Lisheng Qiu

**Affiliations:** 1 Department of Thoracic and Cardiovascular Surgery, Shanghai Children’s Medical Center, School of Medicine, Shanghai Jiao Tong University, Shanghai, China; 2 Department of Pediatric Intensive Care Unit, Shanghai Children’s Medical Center, School of Medicine, Shanghai Jiao tong University, Shanghai, China; 3 Basic Medical School, Shangdong First Medical University, Shangdong, China; 4 Institute of Pediatric Translational Medicine, Shanghai Children’s Medical Center, School of Medicine, Shanghai Jiao Tong University, Shanghai, China; 5 Shanghai Institute for Pediatric Congenital Heart Disease, Shanghai Children’s Medical Center, School of Medicine, Shanghai Jiao Tong University, Shanghai, China

**Keywords:** Experimental models of disease, Disease model

## Abstract

The molecular atlas of postnatal mouse ventricular development has been made available and cardiac regeneration is documented to be a downregulated process. The right ventricle (RV) differs from the left ventricle. How volume overload (VO), a common pathologic state in children with congenital heart disease, affects the downregulated processes of the RV is currently unclear. We created a fistula between the abdominal aorta and inferior vena cava on postnatal day 7 (P7) using a mouse model to induce a prepubertal RV VO. RNAseq analysis of RV (from postnatal day 14 to 21) demonstrated that angiogenesis was the most enriched gene ontology (GO) term in both the sham and VO groups. Regulation of the mitotic cell cycle was the second-most enriched GO term in the VO group but it was not in the list of enriched GO terms in the sham group. In addition, the number of Ki67-positive cardiomyocytes increased approximately 20-fold in the VO group compared to the sham group. The intensity of the vascular endothelial cells also changed dramatically over time in both groups. The Kyoto Encyclopedia of Genes and Genomes (KEGG) pathway analysis of the downregulated transcriptome revealed that the peroxisome proliferators-activated receptor (PPAR) signaling pathway was replaced by the cell cycle in the top-20 enriched KEGG terms because of the VO. Angiogenesis was one of the primary downregulated processes in postnatal RV development, and the cell cycle was reactivated under the influence of VO. The mechanism underlying the effects we observed may be associated with the replacement of the PPAR-signaling pathway with the cell-cycle pathway.

## Introduction

Promoting the proliferation of endogenous cardiomyocytes is an important research direction for heart regeneration and an important means of treating heart failure [1–4]. Both neonatal mouse and human cardiomyocytes possess strong proliferative potential but with increasing age, the proliferative potential of cardiomyocytes gradually disappears, and by adulthood, the proliferative capability is negligible [5–8]. To determine the molecular mechanisms that mediate postnatal loss of cardiomyocyte proliferation, Virpi et al. combined transcriptomics with proteomic and metabolomic analyses in the early postnatal mouse heart (from postnatal day 1[P1] to P23) [9]. Although these authors developed the molecular atlas of postnatal cardiac development and highlighted the importance of metabolic pathways as potential targets for cardiomyocyte proliferation, the downregulated processes involved in postnatal ventricular development remain unclear. As the loss of cardiomyocyte proliferation is a downregulated process that begins from P7 [1–4], analyzing the downregulatory process after P7 may delineate a clearer trajectory of the overall loss of cardiomyocyte proliferation.

Our current understanding of the development of cardiomyocytes after birth is derived from whole-ventricle analysis or left-ventricle analysis, and right-ventricular analysis is lacking [9, 10]. As the left and right ventricles are quite different in their embryologic origins, molecular structures, and anatomical functions, the data derived from the whole ventricle or left ventricle cannot be directly applied to the right ventricle (RV) [11–13]. More importantly, the left and right ventricles react differently when facing the same stimulus. For example, in the face of pressure overload, the proliferation of neonatal cardiomyocytes in the RV is more profound than in the left ventricle [5, 14]. In addition, the treatment and prognosis of many children with congenital heart disease are determined by the functions of the RV as pertaining to left ventricular dysplasia or transposition of the great arteries, for example [15, 16]. Therefore, the analysis of postnatal right-ventricular development is not only useful for basic research but also provides important clinical implications.

Volume overload (VO) is often noted in various types of congenital heart disease in children, including atrial septal defect, repair of tetralogy of Fallot, and pulmonary valve insufficiency [17–19]. We then ask, does VO affect the downregulated development of right-ventricular cardiomyocytes? If so, how? Solving these issues may provide a theoretical basis and targets for the treatment of congenital heart disease.

To fully understand the downregulated process of RV and how VO alters this process, we herein constructed a prepubertal mouse right-ventricular VO model at P7 and followed this up to P21, on which cardiomyocyte is fully matured [3].

## Results

### Generation of RV VO in AVF mice

**Fig. 1:**
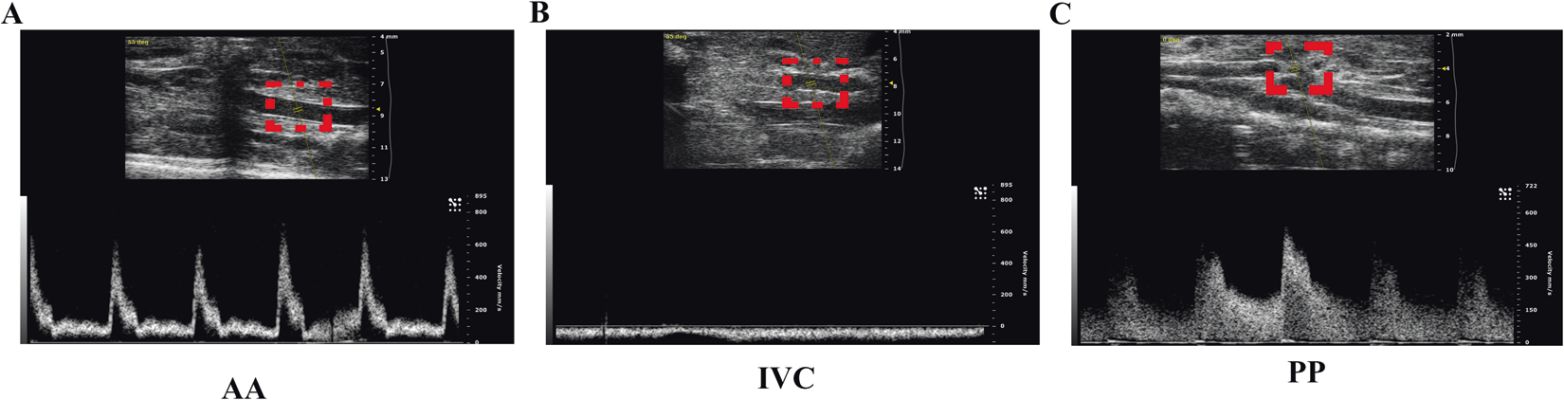
Establishment of the abdominal aorta and inferior vena cava fistula (AVF). **A** There was pulsatile blood flow in the abdominal aorta (AA), with a peak blood flow velocity of 600 mm/s. **B** The inferior vena cava (IVC) manifested no pulsatile blood flow. **C** Representative image of the pulsating blood flow at the puncture point, with a peak blood flow velocity of 580 mm/s.

As shown in Fig. 1A, we observed a pulsatile blood flow in the abdominal aorta (AA), with peak flow velocity up to 600 mm/s but we did not detect any pulsatile blood flow in the inferior vena cava (IVC) (Fig. 1B). At the puncture point (PP), we noted a pulsatile blood flow (Fig. 1C, Supplementary video S1), with a peak flow velocity up to 580 mm/s (Fig. 1C). These results suggested that the creation of a successful fistula between the AA and IVC.

**Fig. 2:**
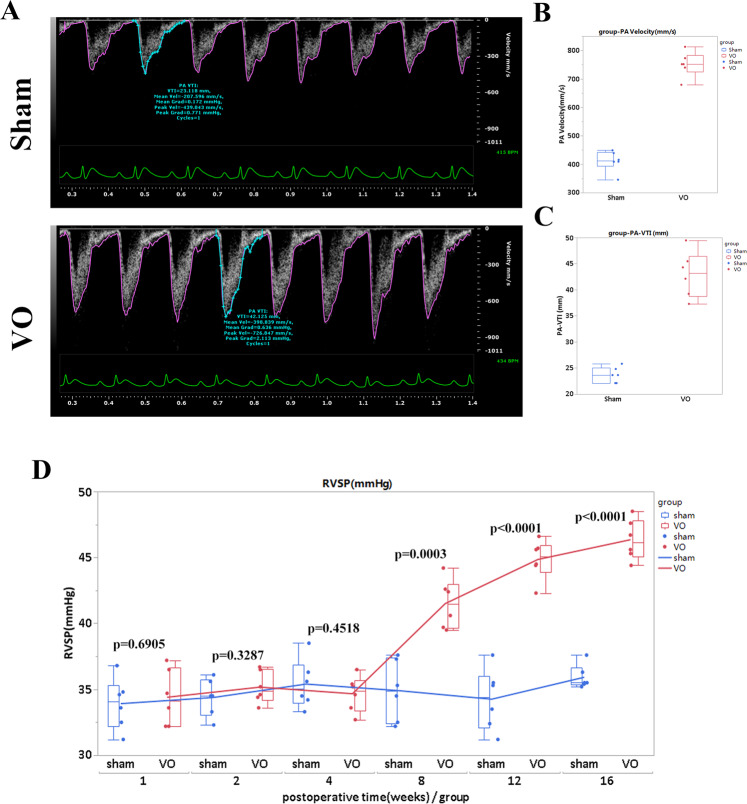
Verification of VO in the AVF group. **A** Representative echocardiogram of pulmonary artery (PA) velocity and velocity-time integral (VTI) in the sham and VO groups. **B** Quantification of PA-velocity in the sham and VO groups, *p* < 0.0001, *N* = 6. **C** Quantification of PA-VTI in the sham and VO groups, *p* < 0.0001, *N* = 6. **D** RVSP changes over time. There were no significant differences between the sham and VO groups before 4 weeks after the AVF operation. RVSP right-ventricular systolic pressure.

To confirm that there was VO in the RV, we examined the pulmonary artery (PA)-velocity and PA-velocity-time integral (VTI) 7 days after the AVF creation. The PA-velocities in the sham and VO groups were 411.3 ± 36.1 and 751.4 ± 43.7 mm/s, respectively (*P* < 0.0001, *n* = 6, Fig. 2A, B); and the PA-VTIs in the sham and VO groups were 23.7 ± 1.5 and 42.9 ± 4.4 mm, respectively (*P* < 0.0001, *n* = 6, Fig. 2A, C). These results thus verified that the RV VO was successfully established. To ascertain whether only VO and not pressure overload occurred during our investigational period (P14–P21), we determined, at the first 4 weeks after the AVF creation, that there were no differences in RV systolic pressure (RVSP) between the sham and VO groups (Fig. 2D); this suggested that during our investigational period (the first 2 weeks after the AVF creation), pressure overload exerted little or no effects on the RV.

### Downregulated processes of postnatal RV development are modified by VO

**Fig. 3:**
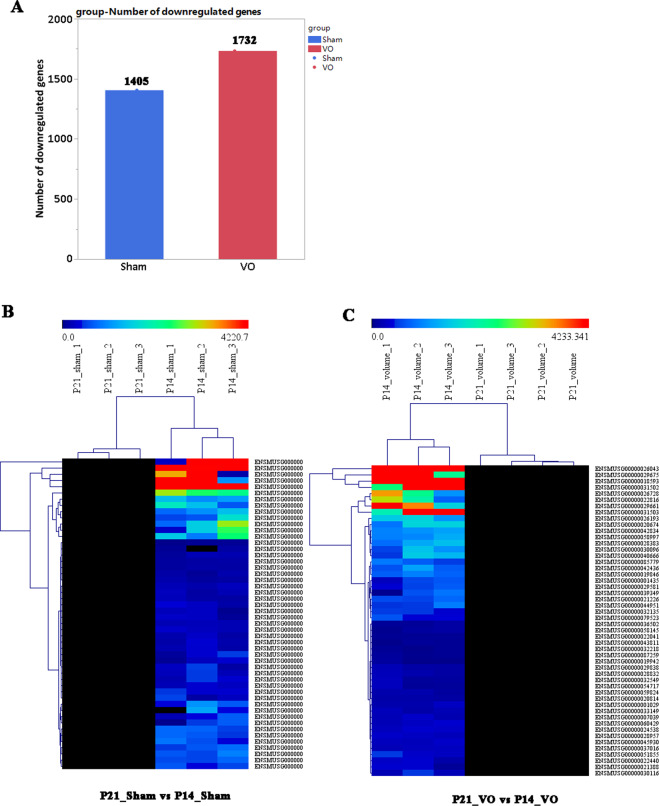
The downregulated transcriptome of postnatal RV development is changed by VO. **A** The number of downregulated genes for postnatal RV development under normal conditions (P21_sham vs. P14_sham) and under the influence of VO (P21_VO and P14_VO). **B** Cluster analysis of the downregulated genes in normal RV development (each group contained three mice). The redder the color, the higher the expression level; the bluer the color, the lower the expression level. The clusters of genes in each group are quite different from each other but similar within the same group. **C** Cluster analysis of the downregulated genes in VO-influenced RV development (each group contained three mice). The redder the color, the higher the expression level; the bluer the color, the lower the expression level. The clusters of genes in each group are quite different from each other but similar within the same group.

To develop a clearer picture of the downregulated process of postnatal RV development (from P14 to P21), we only selected the downregulated transcriptome of the RV for cluster analysis. Our results showed that without VO, there were 1405 downregulated genes from P14 to P21 (Fig. 3A) and that VO increased this number to 1732 (Fig. 3A). When these genes were clustered, a heatmap showed that the individual mice in the same group were similar to each other but differed noticeably from the mice in the other group (Fig. 3B, C). These results indicated that the downregulated processes of postnatal RV development were altered by VO.

### Cell-cycle activities reappear in the biological process of postnatal development of the RV because of VO

**Fig. 4:**
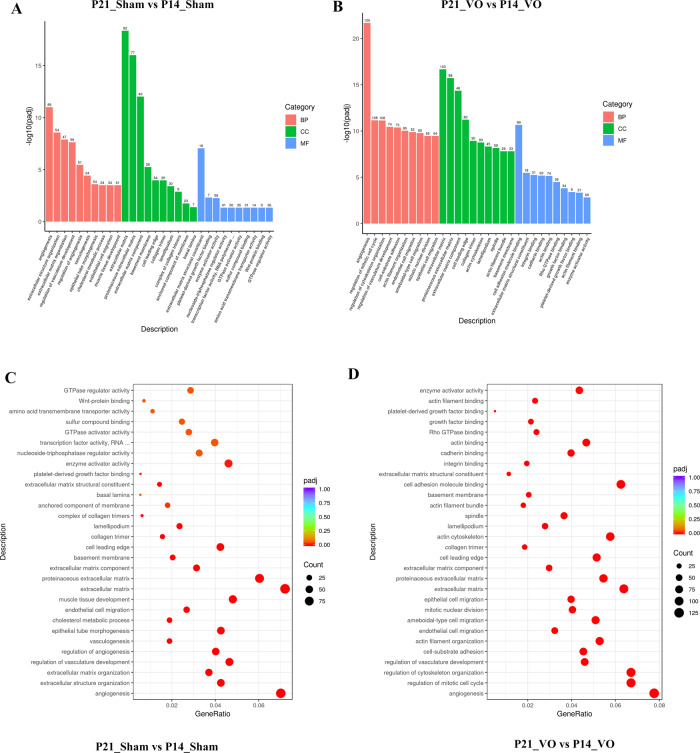
The downregulated biological processes of postnatal RV development are altered by VO. **A** GO analysis of the downregulated transcriptome of RV development. From the results of the GO-enrichment analysis, the 10 most significant terms are displayed. The abscissa is the GO term, and the ordinate is the significance level for GO term enrichment. The higher the value, the more significant the result; and the different colors represent three different GO subclasses: biological process (BP), cellular component (CC), and molecular function (MF). **B** The GO analysis of the downregulated transcriptome of VO-influenced RV development. From the results of the GO-enrichment analysis, the most significant 10 terms are displayed. **C** GO analysis of the downregulated transcriptome of RV development. From the results of the GO-enrichment analysis, we selected the 30 most significant terms to construct scatter plots for display. **D** GO analysis of the downregulated transcriptome of VO-influenced RV development.

Gene ontology (GO) analysis was applied to analyze the downregulated transcriptome. As shown in Fig. 4, during postnatal RV development, angiogenesis was the most enriched GO term in both the sham and VO groups, suggesting that the downregulation of angiogenesis is one of the most important processes of postnatal RV development and that VO does not change this process. The difference between the sham and VO groups is in the regulation of the mitotic cell cycle, which was the second-most enriched GO term in the VO group but was not present in the list of enriched GO terms of the sham group. This result suggested that the cell-cycle activities that reappeared in the biological process of postnatal development of the RV were due to VO.

### The pathways that control the downregulated processes of RV development are changed by VO

**Fig. 5:**
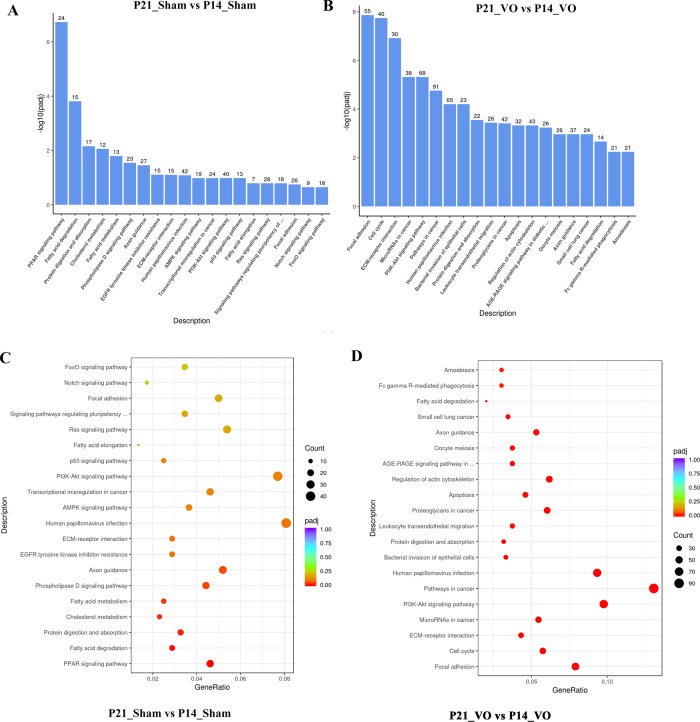
KEGG-pathway analysis reveals an underlying mechanism mediating the alterations in biological processes by VO. **A** Histogram of the 20 most significant KEGG pathways of the downregulated genes in normal RV development. **B** Histogram of the 20 most significant KEGG pathways entailing downregulated genes in the VO-influenced RV development. The 20 most significant KEGG pathways from the KEGG-enrichment results are displayed. The abscissa is the KEGG pathway, and the ordinate is the significance level of pathway enrichment. The higher the value, the greater the significance. **C** Scatterplot of the 20 most significant KEGG pathways in normal RV development. **D** Scatterplot of the 20 most significant KEGG pathways in VO-influenced RV development. The abscissa is the ratio of the number of downregulated genes in the KEGG-pathway analysis to the total number of downregulated genes, the ordinate is the KEGG pathway, the size of the dots represents the number of genes annotated to the KEGG pathway, and the colors from red to purple represent the significance level of KEGG-pathway enrichment.

To understand the underlying mechanism by which VO modified the downregulated processes of postnatal RV development, we applied KEGG-pathway analysis. As shown in Fig. 5, the sham and VO groups shared many enriched terms of the KEGG pathway, such as focal adhesion, ECM-receptor interaction, and PI3K/Akt-signaling pathway, although their sequence was altered by VO. An interesting finding was that the peroxisome proliferators-activated receptor (PPAR) signaling pathway was the most enriched term of the sham group but was not listed among the enriched KEGG terms of the VO group. In contrast, the cell cycle was the second-most enriched term of the VO group but it was not found in the list of enriched KEGG terms of the sham group. This result confirmed that cell-cycle regulation was one of the most important biological processes affected by VO during postnatal development.

### Verification of RNA-seq results by the examination of angiogenesis and cell cycle

To confirm the RNA-seq results, 20 genes manifesting the greatest fold changes were verified by quantitative real-time PCR (qRT-PCR), and angiogenesis and the cell cycle were further analyzed.

**Fig. 6:**
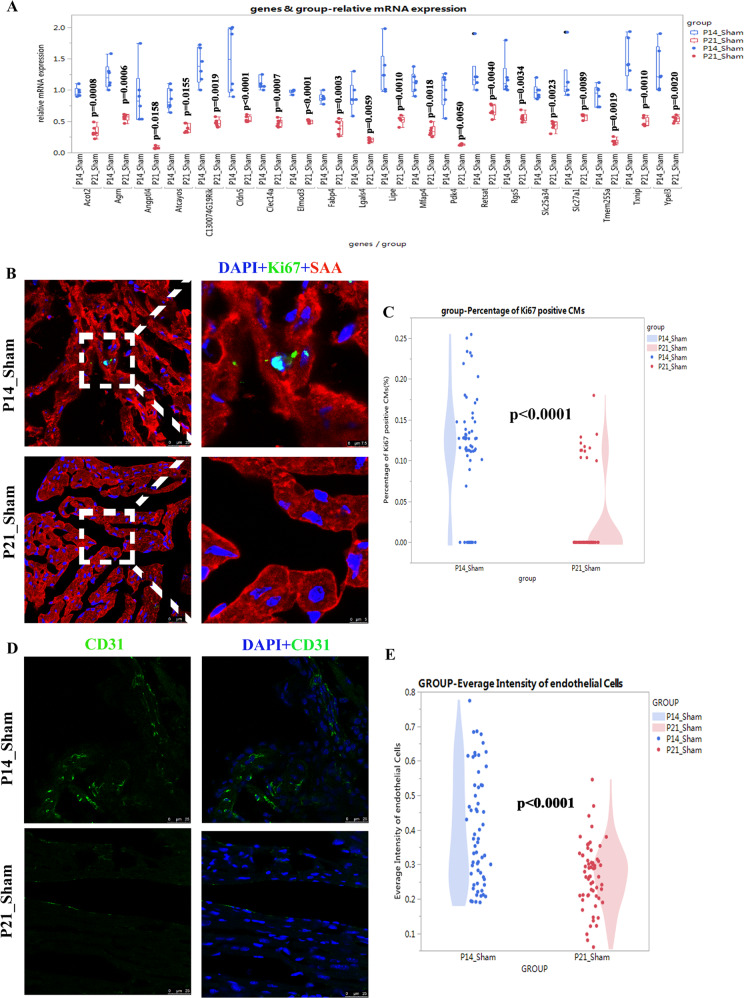
Verification of downregulated processes during postnatal RV development. **A** The 20 genes manifesting the greatest fold changes of the downregulated transcriptome. **B** Representative Ki67-positive cardiomyocytes in the sham group. **C** Quantification of Ki67-positive cardiomyocytes in the sham group (*N* = 60 slides from six mice). **D** Representative CD31-positive cells in the sham group. **E** Quantification of the average intensity of endothelial cells (*N* = 60 slides from six mice).

As shown in Fig. 6A, in the top-20 genes from the RNA-seq data, six (*Mfap4*, *Agrn*, *Pdk4*, *Clec14a*, *Angptl4*, *and Cldn5*) are closely related to angiogenesis. As shown in Fig. 6B and C, Ki67 (a marker of cell-cycle active cells) was present in the cardiomyocytes of the sham group. However, the percentages of Ki67-positive cardiomyocytes were only 0.114 ± 0.069% in the P14_Sham group and 0.024 ± 0.049% in the P21_Sham group, indicating that there were very limited cell-cycle-active cardiomyocytes in the sham group. As shown in Fig. 6D and E, the average intensity of CD31 (a marker of vascular endothelium) was significantly reduced in the P21_Sham group when compared to the P14_Sham group, indicating that angiogenesis was one of the major processes involved in postnatal RV development.

**Fig. 7:**
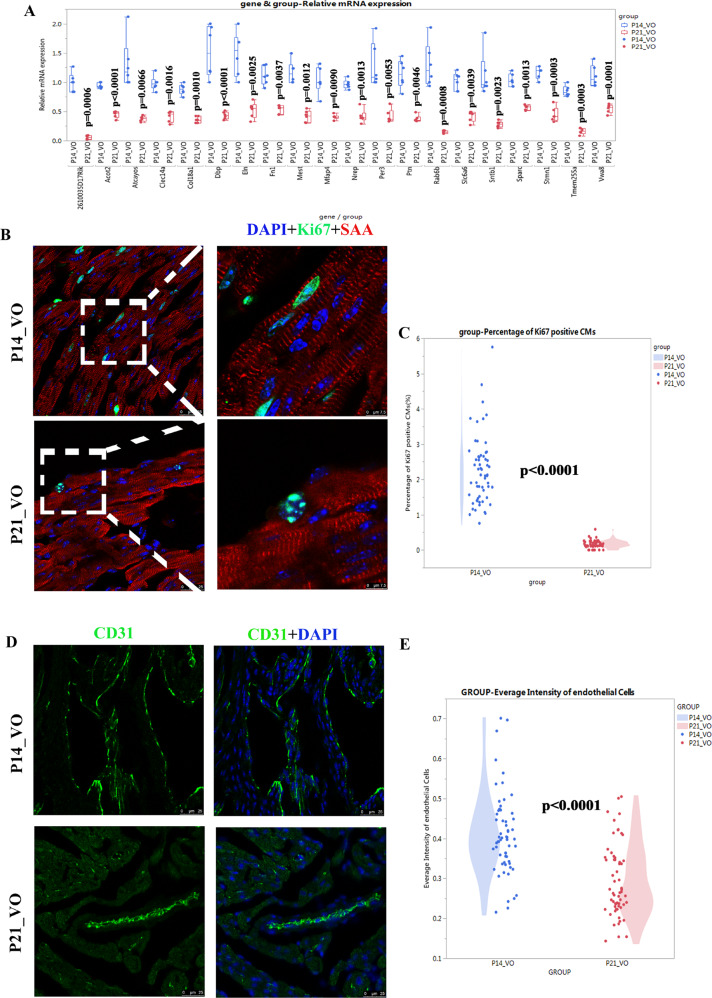
Verification of downregulated processes during VO-influenced postnatal RV development. **A** The 20 genes manifesting the greatest fold changes of the downregulated transcriptome. **B** Representative Ki67-positive cardiomyocytes in the VO group. **C** Quantification of Ki67-positive cardiomyocytes in the VO group. (*N* = 60 slides from six mice). **D** Representative CD31-positive cells in the VO group. **E** Quantification of the average intensity of endothelial cells (*N* = 60 slides from six mice).

As shown in Fig. 7A, of 20-fold genes exhibiting the greatest fold changes of the VO-influenced downregulated transcriptome, five (*Clec14a*, *Col18a1*, *Sparc*, *Fn1*, and *Ptn*) are closely related to angiogenesis. As shown in Fig. 7B and C and Supplementary Fig. S2, the percentages of Ki67-positive cardiomyocytes was up to 2.246 ± 0.955% in the P14_VO group and 0.169 ± 0.107% in the P21_VO group. Compared with the sham group on P14, Ki67-positive cardiomyocytes in VO group on P14 increased approximately 20-fold, confirming that the cell cycle was activated by VO during postnatal RV development. Similarly, the change in CD31 intensity in the VO group was comparable to the sham group (Fig. 7D, E).

Collectively, the above results confirmed that the downregulated processes of postnatal RV development were altered by VO.

## Discussion

In the current study, we demonstrated for the first time that the downregulation of angiogenesis is one of the most important biological processes of postnatal RV development from P7 to P21, and our analysis of the downregulated transcriptome highlighted the importance of angiogenesis in postnatal loss of cardiomyocyte proliferation. Some previous studies have shown that angiogenesis plays an important role in heart regeneration or cardiomyocyte proliferation [14, 20]. For example, Mona et al. showed that pressure overload induced both cardiomyocyte proliferation and angiogenesis in neonatal mice. However, when angiogenesis was inhibited, cardiomyocyte proliferation was blocked accordingly [14]. Our current data showed that from P14 to P21, angiogenesis was the most enriched GO term and that there were 89 genes in this GO term (Fig. 4A). Under the influence of VO, although angiogenesis was still the most enriched GO term, the number of genes in this GO term was upregulated to 125 (Fig. 4B), suggesting that the VO increased the process of angiogenesis. This finding has never before been reported using whole-transcriptome analysis [9, 21] and highlights the importance of downregulated-transcriptome analysis.

We also found that the regulation of the mitotic cell cycle was the second-most enriched GO term in the VO group and that the number of genes in this term was 108 (Fig. 4B), suggesting that under the influence of VO, cardiomyocytes can re-enter the cell cycle. In contradistinction, there were no cell-cycle-associated enriched GO terms in the sham group, further confirming our conclusion that cardiomyocytes had exited from the cell cycle after P7 [22]. The current finding has two implications. One is that the prepubertal VO model may be used to study the underlying molecular mechanisms that drive cardiomyocytes to re-enter the cell cycle; and the other is that in the treatment of children with congenital heart disease, VO may be a beneficial factor, if handled appropriately.

The third significant finding from our study was that VO changes the signaling pathways that modulate the downregulated development of the right ventricle. In the sham group, the PPAR-signaling pathway was the most enriched KEGG term (Fig. 5A) but it was not listed in the enriched KEGG terms of the VO group (Fig. 5B). In contrast, in the VO group, the cell cycle was the second-most enriched KEGG term (Fig. 5B) but it was not listed with the enriched KEGG terms of the sham group (Fig. 5A). As the PPAR-signaling pathway has been suggested to be a central component of the cardiomyocyte maturation network [23, 24], it was not surprising that the PPAR-signaling pathway was the most enriched KEGG term in the sham group as the RV undergoes a maturation process between P14 and P21. Intriguingly, the PPAR-signaling pathway was replaced by the cell-cycle pathway under the influence of VO, which suggests that cardiomyocyte maturation and cardiomyocyte proliferation are two opposing processes [25, 26].

Another critical question is why the cardiomyocyte proliferation reactivated by VO ultimately declines. We posit two possible factors as contributing to this phenomenon. One factor is the cardiomyocyte itself. From P7 to P21, cardiomyocytes undergo some important switches: sarcomere isoform switching (such as Myh7–6 and TnI1–3), metabolic switching (glycolysis to oxidative phosphorylation), and electrophysiologic switching (ion channel type and localization changes, T-tubule formation). During this stage, cardiomyocytes are immature and easily stimulated [3]. The other factor is hormonal regulation. There are two reports showing that thyroid hormone is an important regulator of cardiomyocyte proliferation and maturation [27, 28] but the underlying mechanism(s) requires elucidation.

In summary, in the current study, we first demonstrated that angiogenesis is one of the most important downregulated biological processes in postnatal RV development from P7 to P21, featuring the necessity of applying downregulated transcriptomic analysis; this will be useful in uncovering some important information that may not be ascertained by whole-transcriptome analysis. Second, this study revealed that the cell cycle was reactivated by VO and thus provided a novel platform to allow the study of the molecular mechanisms that drive cardiomyocytes to re-enter the cell cycle. This finding also provided a theoretical basis for using VO appropriately as a beneficial factor for children with congenital heart disease. A limitation to our study was that we only evaluated the RV; whether and how the prepubertal LV is changed by VO is currently unknown. Furthermore, whether an altered LV affects the capabilities of the RV is another question that necessitates exploration.

## Materials and methods

The data generated in this study are available from the corresponding author upon reasonable request. All of the RNA-seq data have been deposited in the GEO database (https://www.ncbi.nlm.nih.gov/geo) with accession number GSE 157396.

All of the primer and reagent information are provided in Supplementary Tables 1 and 2.

### Animal experiments

C57/BL6 neonatal mice were randomized into two groups—VO and control groups at postnatal day 7 (P7) and underwent fistula surgery or sham operation. Briefly, under general anesthesia (4% isoflurane), a midline laparotomy was performed to expose the AA and IVC. A needle (diameter, 0.08 mm) was used to puncture through the AA into the IVC (the size of the needle determined the size of the fistula). After the puncture, a 2-min-hemostatic compression was performed before the abdominal wall was closed and the pain was relieved with local lidocaine treatment.

### Abdominal ultrasonography

The fistula between the AA and IVC (AVF) and the PA flow were analyzed with a Vevo 2100 imaging system (Visual Sonics, Toronto, Ontario, Canada). A pulse-wave mode was used to record the waveform in the IVC.

### Echocardiography

We performed echocardiography to evaluate the RV function of the AVF mice with a Vevo 2100 imaging system (Visual Sonics, Toronto, Ontario, Canada). The VTI of the PA blood flow and PA-velocity were calculated from the mean of three consecutive measurements by two dimensional and pulse Doppler echocardiography. In neonates and children, both pulmonary arterial acceleration time (PAT) and the ratio between the PAT and RV ejection time (RVET) are used as complementary parameters to assess physiological and pathological changes in pulmonary hemodynamics [29]. Regardless of age, body surface area, and heart rate [30], the PAT/RVET-index was used as a parameter to estimate the value of RVSP in mice using the following formula as verified by Thibault et al. [31].

RVSP [mmHg] = −83.7 × PAT/RVET-index + 63.7.

### RNA quantification and qualification of the RV-free wall

After anesthetizing the mice with 1.5% isoflurane, their thoracic cavities were opened to obtain the RV-free wall (Supplementary Fig. S1), which was used for RNA extraction with a PureLink RNA Micro Scale Kit. RNA degradation and contamination were monitored on 1% agarose gels, and we assessed RNA purity using a NanoPhotometer^®^spectrophotometer (IMPLEN, CA, USA). RNA integrity was evaluated using the RNA Nano 6000 assay Kit of the Bioanalyzer 2100 system (Agilent Technologies, CA, USA). We executed RT-PCR with a PrimeScript reagent kit and performed qRT-PCR using SYBR Green Power Premix Kits, according to the manufacturers’ instructions and with a 7900 Fast Real-Time PCR System (Applied Biosystems). The following PCR cycle conditions were used: one cycle at 95 °C for 10 s, followed by 40 cycles of 95 °C for 15 s, and 60 °C for 60 s. The primers were obtained from Generay Biotech Co. Ltd. (Shanghai, China). We then calculated the relative fold change using the ΔΔCt method.

### Library preparation

A total of 1 μg of RNA per sample from the RV-free wall was used as the input material for RNA sample preparation, and sequencing libraries were generated using the NEBNext^®^ Ultra^TM^ RNA Library Prep Kit for Illumina^®^ (NEB, USA) according to the manufacturer’s instructions. The index codes were added to attribute sequences to each sample. Briefly, mRNA was purified from total RNA using poly-T oligo-attached magnetic beads. The fragmentation was carried out using divalent cations under an elevated temperature in a NEB Next First Strand Synthesis Reaction Buffer (5×), and the first-strand cDNA was synthesized using random hexamer primers and M-MuLV Reverse Transcriptase (RNase H ^–^); second-strand cDNA synthesis was subsequently performed using DNA polymerase I and RNase H. Remaining overhangs were converted into blunt ends via exonuclease/polymerase activities, and after adenylation of the 3′ ends of the DNA fragments, NEBNext Adaptors with hairpin loop structures were ligated to prepare the sequences for hybridization. To select cDNA fragments that were preferentially 250–300 bp in length, the library fragments were purified with an AMPure XP system (Beckman Coulter, Beverly, MA, USA). Three microliters of USER Enzyme (NEB, USA) were used with size-selected, adaptor-ligated cDNA at 37 °C for 15 min followed by 5 min at 95 °C. Then, PCR was performed with Phusion High-Fidelity DNA polymerase, Universal PCR primers, and Index (X) Primer. PCR products were ultimately purified (AMPure XP system), and library quality was assessed on an Agilent Bioanalyzer 2100 system.

### Clustering and sequencing

The clustering of the index-coded samples was performed on a cBot Cluster Generation System using a TruSeq PE Cluster Kit v3-cBot-HS (Illumina) according to the manufacturer’s instructions. Sequencing was then performed on an Illumina Novaseq platform to generate 150-bp paired-end reads.

### Quality control, read mapping, and quantification of gene expression levels

Raw data (raw reads) in fastq format were first processed through in-house Perl scripts; and reads containing adapters, reads containing poly-N, and low-quality reads were removed from the raw data to generate clean data (clean reads). All of the downstream analyses were thus based on clean, high-quality data.

The reference genome and gene model annotation files were downloaded from the genome website directly. The index of the reference genome was constructed using Hisat2 v2.0.5, and paired-end clean reads were also aligned to the reference genome using Hisat2 v2.0.5; the number of reads mapped to each gene was counted using featureCounts v1.5.0-p3. The fragments per kilobase of transcript sequence per million base pairs sequenced (FPKM) for each gene were calculated based on the length of the gene and read counts mapped to each gene.

### Differential gene expression analysis

Differential gene expression analysis was performed using the DESeq2 R package (1.16.1). DESeq2 provides statistical routines for determining the downregulated expression in digital gene expression data using a model based on a negative binomial distribution. The resulting *P* values were adjusted using Benjamini and Hochberg’s approach for controlling the false discovery rate. Genes with an adjusted *P* value of <0.05 as determined by DESeq2 were considered to have downregulated expression.

### GO- and KEGG-enrichment analyses of genes showing downregulated expression

GO-enrichment analysis of genes showing the downregulated expression was implemented with the clusterProfiler R package. The GO terms with corrected *P* values under 0.05 were considered to be significantly enriched, and the clusterProfiler R package was used to test the statistical enrichment of genes that were downregulated in KEGG pathways (http://www.genome.jp/kegg/).

### Immunofluorescence

The frozen RVs were sectioned onto slides with 8-µm thickness, washed three times with PBS, fixed with 4% paraformaldehyde for 10 min, permeated with 0.5% Triton X-100 for 15 min, blocked with 10% donkey serum for 30 min, and stained with primary antibodies overnight at 4 °C. After washing the slides three more times, we incubated the sections or cells with secondary antibodies and 4′,6-diamidino-2-phenylindole (DAPI) for 30 min. Three researchers who were blinded to sample identity quantified cellular Ki67 staining via digital thresholding, which included image segmentation and creation of a binary image from a grayscale. We then analyzed the converted binary images using the ImageJ software (NIH, Bethesda, Maryland, USA; Laboratory for Optical and Computational Instrumentation, University of Wisconsin, Madison, WI, USA).

### Statistical analysis

We executed statistical analyses were performed using SAS software version 9.2 (SAS Institute Inc., Cary, NC, USA). Continuous data are expressed as means ± one standard deviation. We analyzed differences were tested with the Student’s *t* test when the data were normally distributed; otherwise, data were tested with the rank-sum test. *P* values < 0.05 were considered to be statistically significant.

## Supplementary information

Supplemental Video S1

Supplemental Table S1

Supplemental Table S2

Supplemental Fig.S1

Supplemental Fig.S2
