# Supplementary material for: Downregulated developmental processes in the postnatal right ventricle under the influence of a volume overload
Source: Cell Death Discov. 2021 Aug 7;7:208. doi: 10.1038/s41420-021-00593-y (PMC8349357; doi:10.1038/s41420-021-00593-y)
Supplement: Supplementary file 2 — Supplemental Table S1 [file 41420_2021_593_MOESM2_ESM.docx]

Supplemental Table 1 Primer information

| Gene |  | Sequence (5'->3') |
| --- | --- | --- |
| Lgals4 | Forward | GGTCGTGGTGAACGGAAATTC |
|  | Reverse | GTGGAGGGTTGTACCCAGGA |
| Slc25a34 | Forward | ATGAAGCCAACTCAGGCACAG |
|  | Reverse | GCTGCAACAGAAGACACAAAG |
| Mfap4 | Forward | GGCGTGTATCTCATCTACCCC |
|  | Reverse | TCACTGAGCCGTTGAATCTTTT |
| Agrn | Forward | GCGGTACTTGAAAGGCAAAGA |
|  | Reverse | CTCCAAAGCCACCAATTACCA |
| Pdk4 | Forward | AGGGAGGTCGAGCTGTTCTC |
|  | Reverse | GGAGTGTTCACTAAGCGGTCA |
| Txnip | Forward | GGCCGGACGGGTAATAGTG |
|  | Reverse | AGCGCAAGTAGTCCAAAGTCT |
| C130074G19Rik | Forward | AGAAGTACGGCAAGAATTTTGGG |
|  | Reverse | CCAATGACAATGTACCGGCAT |
| Clec14a | Forward | GTTGCACCCGGACTCAGAAG |
|  | Reverse | GCACAGAACCTCAAACTGGTACT |
| Lipe | Forward | GATTTACGCACGATGACACAGT |
|  | Reverse | ACCTGCAAAGACATTAGACAGC |
| Ypel3 | Forward | GCGGATTTCAAAGCCCAAGAC |
|  | Reverse | GAGATGAGGTCGTCGTGGTTG |
| Slc27a1 | Forward | CTGGGACTTCCGTGGACCT |
|  | Reverse | TCTTGCAGACGATACGCAGAA |
| Atcayos | Forward | ACTGTGTCCTACTGGATCTTCAA |
|  | Reverse | CAGGTTCCTACTCGTGGCA |
| Rgs5 | Forward | CGCACTCATGCCTGGAAAG |
|  | Reverse | TGAAGCTGGCAAATCCATAGC |
| Acot2 | Forward | GTTGTGCCAACAGGATTGGAA |
|  | Reverse | GCTCAGCGTCGCATTTGTC |
| Angptl4 | Forward | CATCCTGGGACGAGATGAACT |
|  | Reverse | TGACAAGCGTTACCACAGGC |
| Tmem255a | Forward | TGGGAGCCTTCAATAGGAGGA |
|  | Reverse | ACCGTGAGAATTAACATGGACAC |
| Retsat | Forward | GTCTACGTGGGCCTTTACGC |
|  | Reverse | ACTTTCTTCCTAGCCTCCTTGTC |
| Elmod3 | Forward | TTCAAGATGGGCAGCTAGAAAAT |
|  | Reverse | TCGTCAGCTCGGAGATAGGAA |
| Fabp4 | Forward | AAGGTGAAGAGCATCATAACCCT |
|  | Reverse | TCACGCCTTTCATAACACATTCC |
| Cldn5 | Forward | GCAAGGTGTATGAATCTGTGCT |
|  | Reverse | GTCAAGGTAACAAAGAGTGCCA |
| Per3 | Forward | AACACGAAGACCGAAACAGAAT |
|  | Reverse | CTCGGCTGGGAAATACTTTTTCA |
| Rab6b | Forward | AACCCGCTGCGAAAATTCAAG |
|  | Reverse | CGGTCTTCCAAGTACATGGTTT |
| Nrep | Forward | AATTCTCTTCGACCCCAAAACTT |
|  | Reverse | CGTCAGTCCGTCTCTATCGTT |
| Sntb1 | Forward | AGCGGACTGCTGGAAGTTTT |
|  | Reverse | GCAGAACGAACCATTGGTGG |
| Mest | Forward | AGAGTGGTGGGTCCAAGTAGG |
|  | Reverse | AAGCACAACTATCTCAGGGCT |
| 2610035D17Rik | Forward | GGAGAGCGCACTCCATGAC |
|  | Reverse | CGCAGCTCAGCATATAGGTCC |
| Slc6a6 | Forward | GCGTTTCCCGTACCTCTGC |
|  | Reverse | ATGGATGCGTAGCCAATGCC |
| Col18a1 | Forward | GTGCCCATCGTCAACCTGAA |
|  | Reverse | GACATCTCTGCCGTCAAAAGAA |
| Sparc | Forward | TGGGAGAATTTGAGGACGGTG |
|  | Reverse | GAGTCGAAGGTCTTGTTGTCAT |
| Fn1 | Forward | ATGTGGACCCCTCCTGATAGT |
|  | Reverse | GCCCAGTGATTTCAGCAAAGG |
| Ptn | Forward | ATGTCGTCCCAGCAATATCAGC |
|  | Reverse | CCAAGATGAAAATCAATGCCAGG |
| Dbp | Forward | GGAAACAGCAAGCCCAAAGAA |
|  | Reverse | CAGCGGCGCAAAAAGACTC |
| Stmn1 | Forward | TCTGTCCCCGATTTCCCCC |
|  | Reverse | AGCTGCTTCAAGACTTCCGC |
| Vwa8 | Forward | GAGATGTGTCCTACAAGCTGAAA |
|  | Reverse | CTTGACAGGGCGATGTATTCC |
| Eln | Forward | TGTCCCACTGGGTTATCCCAT |
|  | Reverse | CAGCTACTCCATAGGGCAATTTC |
