# Supplementary material for: Downregulated developmental processes in the postnatal right ventricle under the influence of a volume overload
Source: Cell Death Discov. 2021 Aug 7;7:208. doi: 10.1038/s41420-021-00593-y (PMC8349357; doi:10.1038/s41420-021-00593-y)
Supplement: Supplementary file 3 — Supplemental Table S2 [file 41420_2021_593_MOESM3_ESM.docx]

Supplemental Table 2 Reagents

| Name | Company | Catalog No. |
| --- | --- | --- |
| Triton X-100 | Sigma-Aldrich | T9284 |
| Hematoxylin and eosin Kit | Beyotime biotech | C0105M |
| DNase | Worthington, Lakewood, NJ, USA | 9003-98-9 |
| RNase | Worthington, Lakewood, NJ, USA | 9001-99-4 |
| paraformaldehyde(PFA) | Sigma-Aldrich | 158127 |
| 4',6-diamidino-2-phenylindole(DAPI) | ThermoFisher Scientific | D3571 |
| CD31 | Abcam | ab222783 |
| Ki67 | Abcam | ab15580 |
| PureLink RNA Micro Scale Kit | Life Technologies, Carlsbad, California, USA | 12183016 |
| PrimeScriptTM reagent kit | Takara Bio, Kusatsu, Japan | RR037A |
| SYBR Green Power Premix Kits | Applied Biosystems, Foster City, California | 4368577 |
| NEB Next® UltraTM RNA Library Prep Kit | NEB, USA | E7760 |
| TruSeq PE Cluster Kit | Illumina | v3-cBot-HS |
