# Supplementary figures and images for: Downregulated developmental processes in the postnatal right ventricle under the influence of a volume overload
Source: Cell Death Discov. 2021 Aug 7;7:208. doi: 10.1038/s41420-021-00593-y (PMC8349357; doi:10.1038/s41420-021-00593-y)

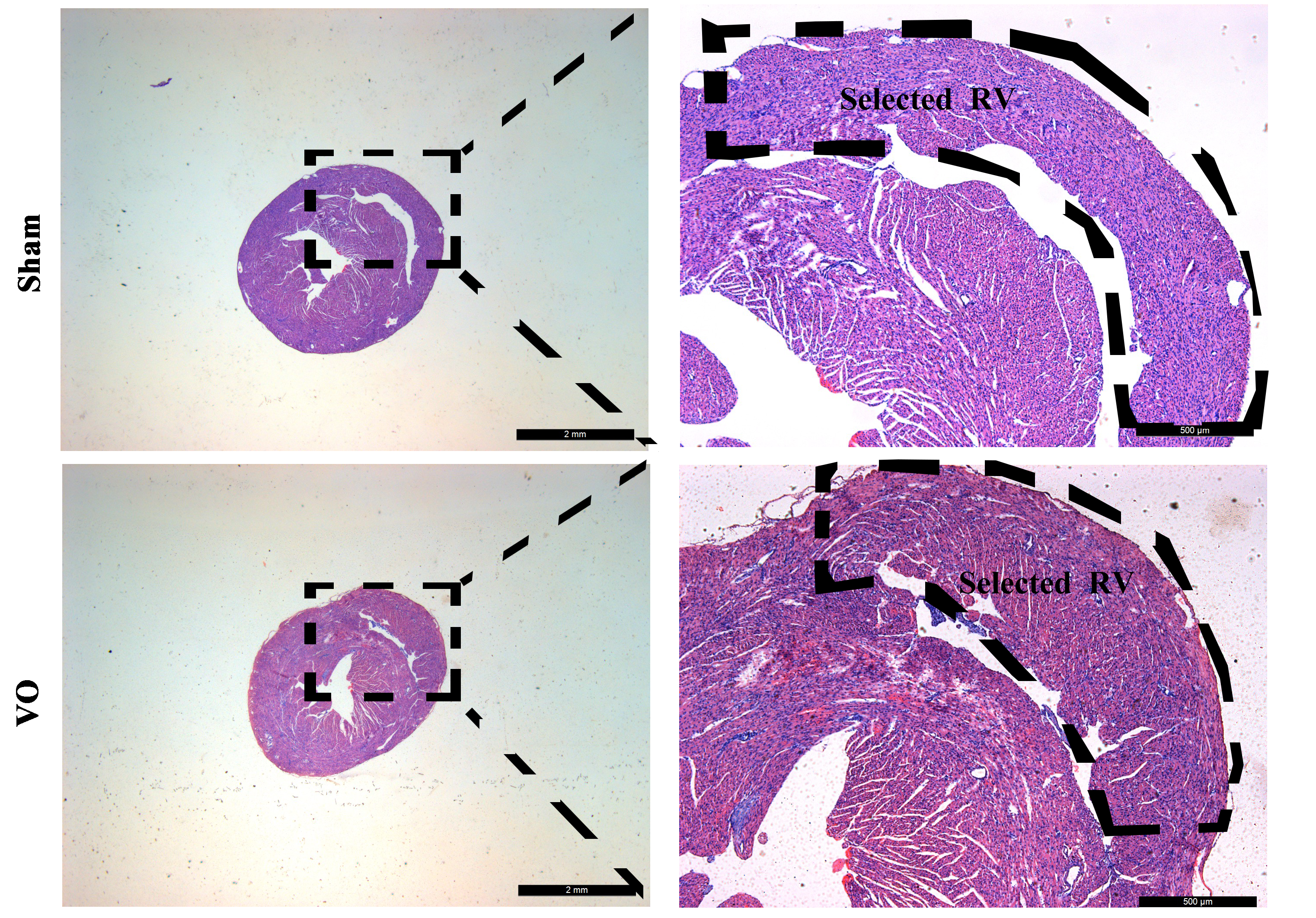

Supplement: Supplementary file 4 — Supplemental Fig.S1 [file 41420_2021_593_MOESM4_ESM.tif]

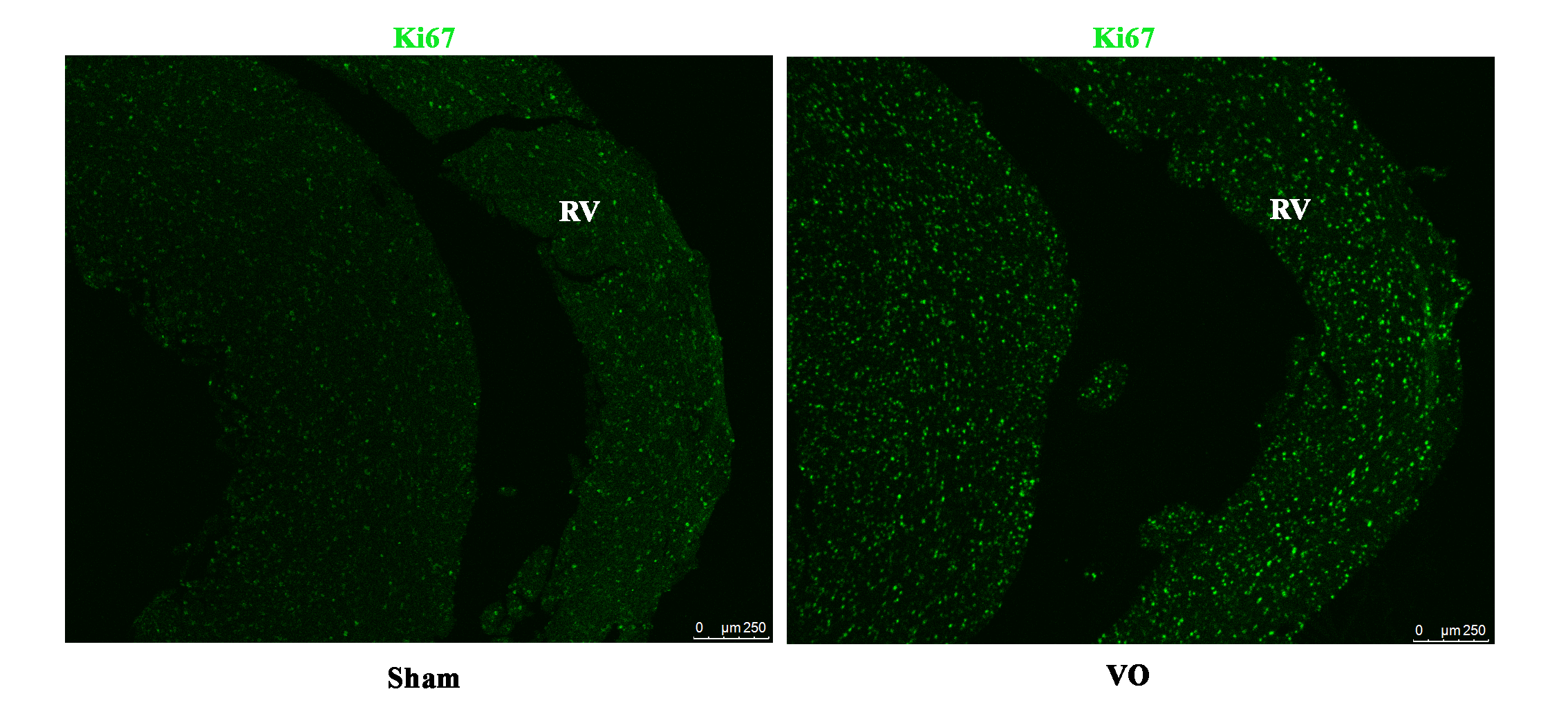

Supplement: Supplementary file 5 — Supplemental Fig.S2 [file 41420_2021_593_MOESM5_ESM.tif]
